# FAM3B mediates high glucose-induced vascular smooth muscle cell proliferation and migration via inhibition of miR-322-5p

**DOI:** 10.1038/s41598-017-02683-3

**Published:** 2017-05-23

**Authors:** Wenxiang Zhang, Siyu Chen, Zhao Zhang, Chen Wang, Chang Liu

**Affiliations:** 1 School of Life Sciences, China Pharmaceutical University, Nanjing, Jiangsu China; 2 Jiangsu Key Laboratory of of Drug Discovery for Metabolic Disease and State Key Laboratory of Natural Medicines, China Pharmaceutical University, Nanjing, Jiangsu China; 3 School of Basic Medical Sciences and Jiangsu Key Laboratory of Human Functional Genomics, Nanjing Medical University, Nanjing, Jiangsu China; 4 Jiangsu Key Laboratory for Molecular and Medical Biotechnology and College of Life Sciences, Nanjing Normal University, Nanjing, Jiangsu China

## Abstract

The proliferation and migration of vascular smooth muscle cells (VSMCs) play an essential role during the development of cardiovascular diseases (CVDs). While many factors potentially contribute to the abnormal activation of VSMCs, hyperglycemia is generally believed to be a major causative factor. On the other hand, FAM3B (named PANDER for its secretory form) is a uniquely structured protein strongly expressed within and secreted from the endocrine pancreas. FAM3B is co-secreted with insulin from the β-cell upon glucose stimulation and regulates glucose homeostasis. In the present study, we sought to determine the roles of FAM3B in the regulation of VSMC physiology, especially under the hyperglycemic condition. We found that FAM3B expression was induced by hyperglycemia both *in vivo* and *in vitro*. FAM3B knockdown inhibited, whereas FAM3B overexpression accelerated VSMC proliferation and migration. At the molecular level, FAM3B inhibited miR-322-5p expression, and enforced expression of miR-322-5p antagonized FAM3B-induced VSMC proliferation and migration, suggesting that FAM3B facilitated VSMC pathological activation via miR-322-5p. Taken together, FAM3B mediates high glucose-induced VSMC proliferation and migration via inhibition of miR-322-5p. Thus, FAM3B may therefore serve as a novel therapeutic target for diabetes-related CVDs.

## Introduction

As is known, vascular smooth muscle cells (VSMCs) are plastic to change their morphology and the rates of proliferation and migration, thus perform contractile and synthetic functions. Under physiological condition, VSMCs stay in an organized, differentiated and contractile phenotype. However, in response to extracellular stimuli, this type of the cells may be switched to a proliferative and migratory phenotype, which is important for wound healing and growth of new vessels in developing tissues^1,2^. Therefore, VSMCs may contribute to the pathogenesis of cardiovascular diseases (CVDs) such as atherosclerosis and restenosis when they are inappropriately activated to proliferate and migrate^3,4^. A wide variety of mitogenic factors have been identified to regulate VSMC proliferation and migration, including nutrients, growth factors, and inflammatory cytokines, through the activation of intracellular signal transduction pathways^5–7^. Among which, the phosphorylation of the extracellular signal regulated kinase (ERK) 1/2 and the progression of cell cycle are the common convergent points for the mitogenic signaling cascades^8^. In this sense, inhibition of VSMC proliferation and migration has high value in the prevention and attenuation of related CVDs.

While many factors potentially contribute to the development of atherosclerosis including abnormalities in plasma lipoproteins and blood pressure seen in diabetic patients, hyperglycemia has been recognized to be a major causative factor^9^. The effect of high glucose levels on the risk of cardiovascular events starts at concentrations below the non-diabetic glucose range (<6.1 mM, and continues to act within the diabetic glucose range (>11.1 mM) in an exponential fashion^10^. High glucose levels regulate structural and functional changes in the vessels involved in diabetic atherosclerosis by modulating several signal transduction pathways^11^. For example, high glucose activates the expression of genes participating in extracellular signal-regulated kinase (ERK)-dependent mitogenic response, and also enhances generation of reactive oxygen species (ROS) products, both of which can stimulate the proliferation and migration of VSMCs^11–13^. In addition, high glucose inhibits apoptosis of VSMCs by up-regulating anti-apoptotic proteins, including Bcl-2, Bcl-xL, and Bfl-1/A1^14,15^. In contrast, the activation of AMPK, a physiological sensor of cellular energy metabolism which has been shown to be able to improve endothelial function and suppress VSMC proliferation and migration, is impaired in high glucose-exposed VSMCs^16^.

FAM3B (named PANDER for its secretory form) is a 235-amino-acid protein that belongs to the family with sequence similarity 3 (FAM3) gene. This family has four members described as FAM3A-D^17^. FAM3B is robustly expressed in the β-cells of islets and co-localizes/co-secretes with insulin granules^17,18^. FAM3B can be sensitively regulated by glucose. Upon glucose stimulation, the secretion of cleaved FAM3B and production of full-length FAM3B are increased in both insulinoma cells and primary islets^19,20^; the promoter activity and mRNA expression levels of *FAM3B* are up-regulated^21^. Glucose induces FAM3B gene expression via multiple signaling pathways that include Ca^2+^ protein kinase A (PKA), Ca^2+^ protein kinase C (PKC), ERK1/2, and cAMP-responsive element-binding protein (CREB) mechanisms. In addition, both phosphoinositide 3- kinase (PI3K)- and ROS-related pathways are involved as well^20^. Conversely, FAM3B acts profoundly in the regulation of glucose homeostasis. Previous studies have shown that FAM3B is critical to maintain normal pancreatic β-cell function and to induce hepatic gluconeogenic gene expression and glucose output during starvation^18,22–25^.

Although FAM3B undoubtedly plays an important role in the glucose homeostasis, it remains unknown whether it affects VSMC pathophysiological behaviors, especially the cell proliferation and migration, under hyperglycemic condition. Therefore, we characterized the expression and function of FAM3B in glucose-challenged rat cultured VSMCs. We demonstrated for the first time that in response to glucose, FAM3B facilitates VSMC proliferation and migration, at least in part, by miR-322-5p inhibition.

## Results

### Hyperglycemia induces FAM3B expression in the VSMC layer of rat thoracic aortas

**Figure 1:**
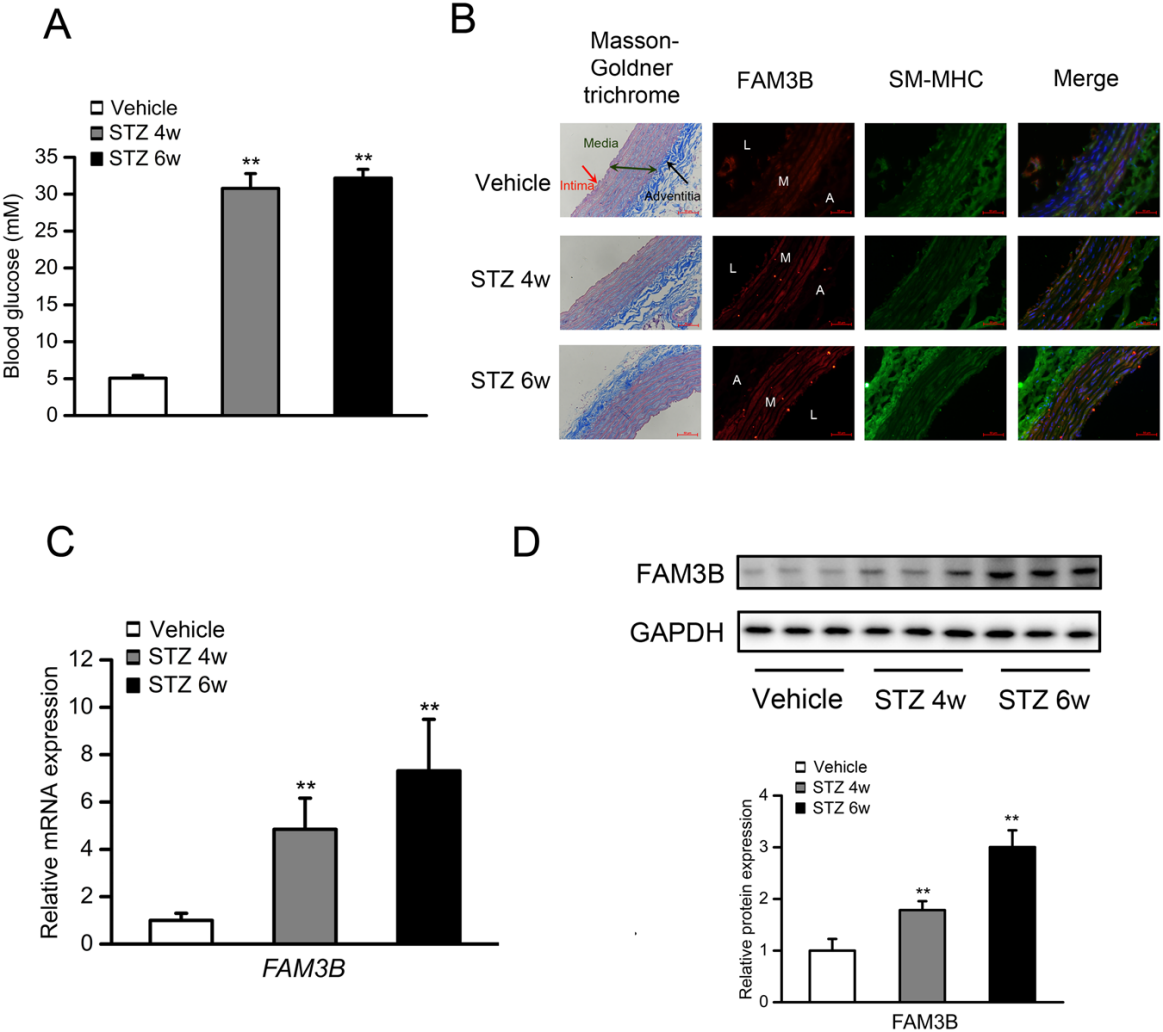
Hyperglycemia induces FAM3B expression in the VSMC layer of rat thoracic aortas. Male SD rats were intraperitoneally injected with STZ at the dose of 55 mg/kg body mass. Control rats were received with equal amount of vehicle (sodium citrate solution). N = 7. (**A**) Blood glucose levels measured in 4 and 6 weeks after STZ injection. (**B**) Masson-Goldner trichrome and immunofluorescence staining of the sections of thoracic aortas isolated from control and hyperglycemic rats. For the Masson-Goldner trichrome staining, blue: collagen fibers; red: muscle fibers. For the immunofluorescence staining, green: SM-MHC; red: FAM3B; blue: DAPI. L: lumen; M: media; A: adventitia. (**C**) RT-qPCR analyses of *FAM3B* mRNA expression levels in the homogenates of VSMC layer dissected from the rat thoracic aorta. (**D**) Western blotting analyses of FAM3B protein expression levels. For all the Western blot images throughout our study, a representative image is shown from at least three separate experiments. ^**^ *P* < 0.01 *vs*. Vehicle. Data are represented as mean ± SD, the same below.

In response to STZ injection, all rats developed hyperglycemia, which was characterized by the increased levels of blood glucose (30.78 mM, 4 weeks post STZ injection and 32.18 mM, 6 weeks post STZ injection) (Fig. 1A). We firstly detected the distribution of FAM3B in the thoracic aorta of normal and hyperglycemic rats. As shown in Fig. 1B, Masson-Goldner trichrome staining indicated that the vessel wall in a healthy artery comprises 3 layers: an outer layer of connective tissue (adventitia where collagen fibers (blue) are enriched in), the medial layer of VSMCs (media where muscle fibers (red) are enriched in) and an inner monolayer of endothelial cells (intima). We next sought to determine the localization of FAM3B by using double staining with antibodies against FAM3B and SM-MHC, a specific marker of VSMC cells. The immunofluorescence results clearly showed a perfect co-localization of SM-MHC (green areas) and FAM3B (red areas) in the VSMC layers (as evidenced by the merged yellow signals). On the other hand, we noticed that green fluorescence signals also appeared in the adventitia, not just limited in the VSMC layers. This may due to the unpredictable cross reaction of the primary antibody of SM-MHC with a non-muscle isoform of MHC^2^. Interestingly, FAM3B protein was significantly increased in the VSMC layer under hyperglycemic condition. Coincided with observations of the immunofluorescence staining, RT-qPCR analysis indicated that the mRNA expression levels of *FAM3B* in the VSMC layer was increased in a time-dependent manner when rats developed hyperglycemia (Fig. 1C). The protein expression levels of FAM3B in the VSMC layer showed a similar trend (Fig. 1D). We also performed immunofluorescence assays to examine the changes of FAM3B expression in the VSMC layer of thoracic aorta in hyperglycemic *db*/*db* mice. In these experiments, we used α-SMA as another marker protein for VSMC cells. As shown in Fig. S1A, α-SMA was positively expressed in the tissue sections we have examined. Meanwhile, the signals of FAM3B (dark green areas dispersively surrounding the nuclei (blue) where the arrows indicate) was much stronger in the same tissue sections dissected from type 2 diabetic *db*/*db* mice. Therefore, the expression of FAM3B was consistently induced in the VSMC layer of thoracic aorta in both STZ-induced and genetically hyperglycemic animals.

### Glucose induces FAM3B expression in VSMCs

**Figure 2:**
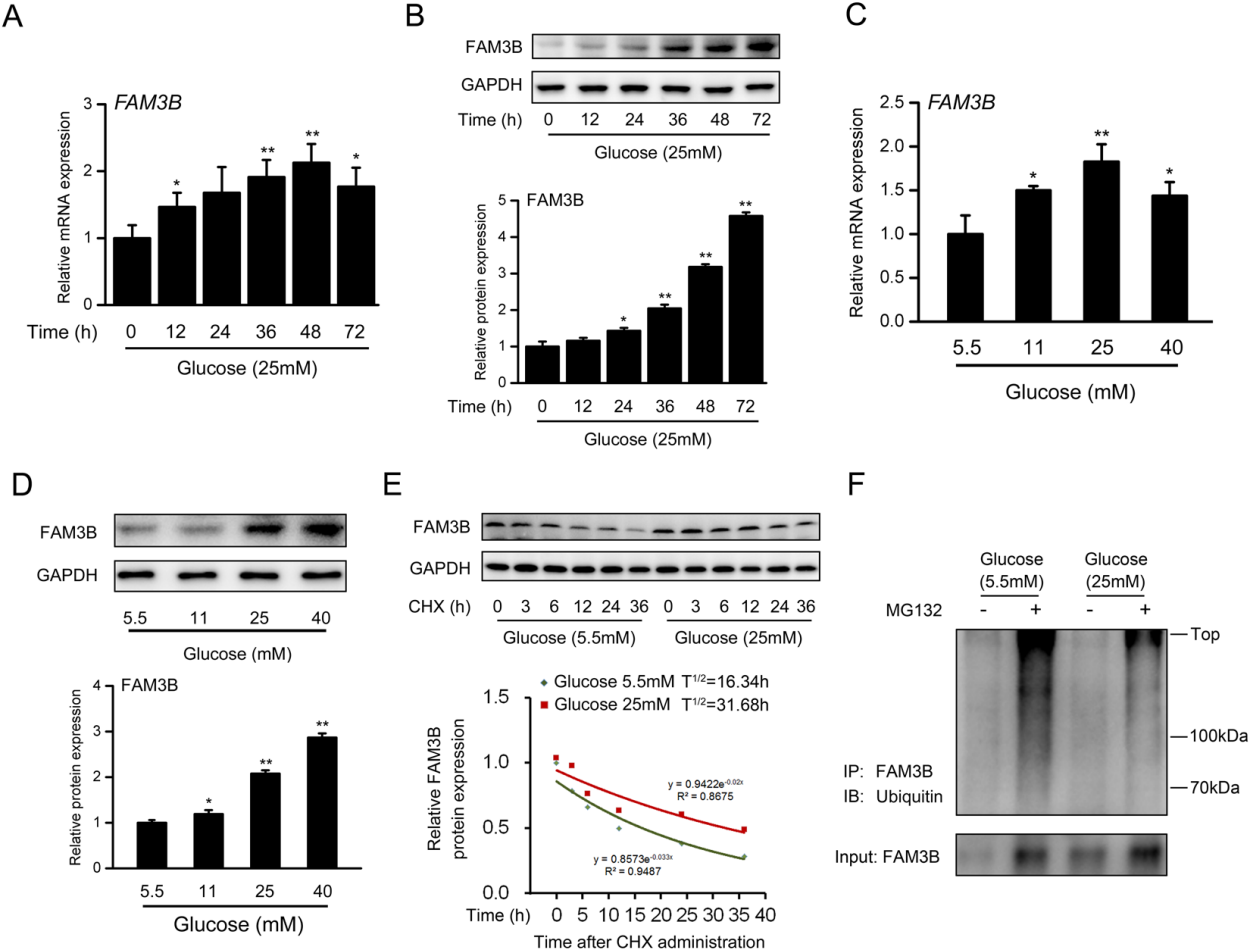
Glucose induces FAM3B expression in VSMCs. Cultured rat VSMCs were stimulated with 25 mM glucose for the indicated time-points. (**A**) RT-qPCR and (**B**) Western blotting analyses of FAM3B expression levels. ^*^ *P* < 0.05 and ^**^ *P* < 0.01 *vs*. 0 h. Cultured VSMCs were stimulated with indicated concentrations of glucose for 36 h. (**C**) RT-qPCR and (**D**) Western blotting analyses of FAM3B expression levels. ^*^ *P* < 0.05 and ^**^ *P* < 0.01 *vs*. 5.5 mM glucose. (**E**) Quantification of the half-life of FAM3B protein by a CHX chase experiment. (**F**) Ubiquitination of FAM3B protein.

As shown in Fig. 2A, 25 mM glucose robustly induced *FAM3B* mRNA expression, which peaked at 48 h and then declined thereafter. FAM3B protein expression was also increased by high glucose in a time-dependent manner (Fig. 2B). We also challenged cultured VSMCs for 24 h with free fatty acids (FFAs) or insulin, two important factors involved in the metabolic homeostasis, to verify their roles in the regulation of FAM3B expression. Interestingly, either 0.4 mM FFAs (an equal molar mixture of oleic acid and palmitic acid) or 100 nM insulin showed modest effects on the FAM3B expression at both transcriptional and translational levels (Fig. S1B,C). This finding suggested that compared with FFAs and insulin, glucose is a more dominate factor to regulate FAM3B expression in VSMCs. In addition to increase FAM3B expression, high glucose inhibited the protein expression levels of contractile marker genes including SM-MHC, SMA, SM22a and SM-calponin (Fig. S2A,B), suggesting that high glucose caused a contractile-to-synthetic phenotypic switch in VSMCs. For the dose-response relationship, we found that FAM3B protein levels were steadily increased by high glucose at all examined doses even when its mRNA levels already declined (e.g. 40 mM glucose) (Fig. 2C,D), implicating a potential post-translational modification for the FAM3B protein. Therefore, we investigated the turnover of FAM3B protein with a cycloheximide (CHX) chase experiment. As shown in Fig. S3A,B, with a prolonged treatment of CHX, the protein expression levels of Cyclin E (T^1/2^ = 16.06 h) and Cyclin D1 (T^1/2^ = 5.95 h) was gradually decreased. In contrast, high glucose stabilized these proteins and extended their half-life times to 29.04 h and 11.18 h, respectively. More importantly, the half-life of FAM3B protein was 16.34 h and 31.68 h for VSMCs cultured in the medium containing low (5.5 mM) and high (25 mM) glucose, respectively (Fig. 2E). Our results indicated that the protein stability of FAM3B was dramatically enhanced in high glucose-treated VSMCs. Accordingly, the ubiquitination of FAM3B protein was dramatically reduced upon high glucose stimulation in the presence of proteasome inhibitor MG132 (Fig. 2F), suggesting that high glucose prevents FAM3B protein degradation.

### Knockdown of FAM3B inhibits high glucose-induced VSMC proliferation and migration

To investigate the essential role of FAM3B in triggering VSMC proliferation and migration, we isolated VSMCs from STZ-induced hyperglycemic rats (we named them H-VSMCs). Consistent with previously reports^26^, these VSMCs exhibited accelerated proliferation and migration compared with those cells isolated from normal rats (we named them N-VSMCs), when assessed by EdU incorporation and wound-healing assays (Fig. S4A,B). At the molecular level, the protein expressions of Cyclin E, proliferative marker protein PCNA and adhesion-associated proteins (VCAM-1 and MMP-9) were significantly induced. Notably, the FAM3B protein was also increased (Fig. S4C). In contrast, the protein expression levels of contractile marker genes were significantly decreased in H-VSMCs (Fig. S4D).

Since hyperglycemia increased both VSMC proliferation/migration rates and FAM3B expression, we next knocked down FAM3B expression by transfecting FAM3B siRNA into H-VSMCs (the knockdown efficiency was validated and shown in Fig. S5A) and observed VSMC behavior. EdU incorporation and directly cell counting assays indicated that knockdown of FAM3B significantly reduced the proliferation rate of H-VSMCs (Fig. S5B–D). As is known, cellular proliferation is tightly associated with cell cycle progression. We thus used flow cytometry analysis to investigate how FAM3B affects cell cycle phases of VSMCs. We found that knockdown of FAM3B in H-VSMCs increased the percentage of cells quiescent at the G0/G1 phase (increased from 84.35 ± 0.77% to 91.15 ± 0.68%) and inhibited S-phase re-entry (decreased from 10.32 ± 0.96% to 3.83 ± 0.4%) (Fig. S5E). At the molecular level, cell cycle progression is finely controlled by cyclins and cyclin-dependent kinases (CDKs). Western blot analysis found that knockdown of FAM3B reduced the protein expression levels of Cyclin E/D1, CDK2/6 and PCNA in H-VSMCs (Fig. S5F). In contrast, the expression of p27, a negative regulator of cyclin-CDK complexes, was increased by FAM3B knockdown.

Next, we performed transwell chamber and wound-healing assays to examine the effect of FAM3B knockdown on VSMC migration. Similar to the proliferation results, FAM3B knockdown inhibited cell migration of H-VSMCs (Fig. S5G–I). Moreover, ICAM-1, VCAM-1 and MMPs are key regulators involved in the cell migration and adhesion^3,7^. As shown in Fig. S5J, knockdown of FAM3B significantly inhibited the protein expression levels of all these proteins. The total activity of MMP-9 and MMP-2 in H-VSMCs was also decreased when FAM3B was knocked down (Fig. S5K,L).

**Figure 3:**
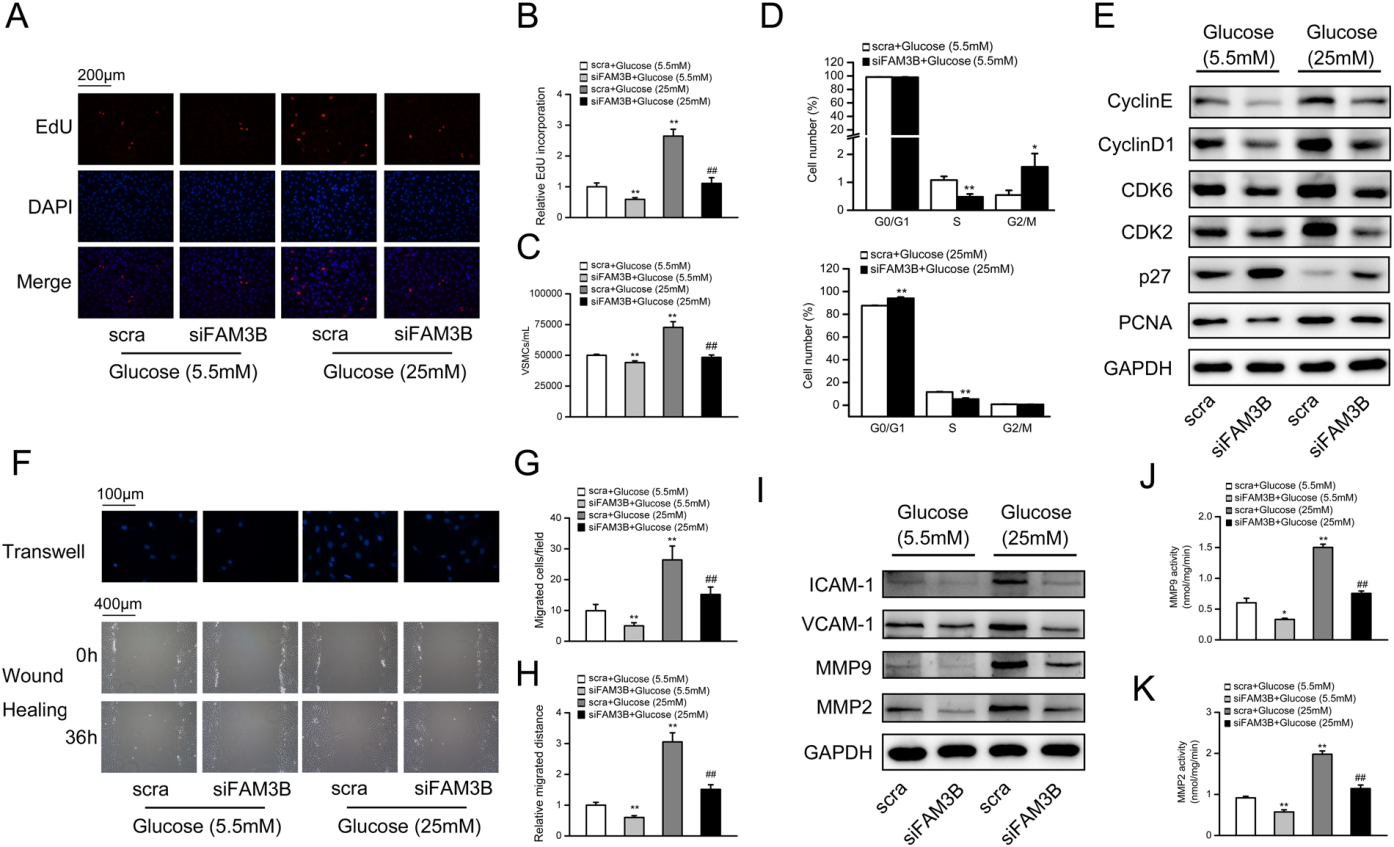
Knockdown of FAM3B inhibits high glucose-induced VSMC proliferation and migration. VSMCs were transfected with scramble (scra) siRNA (as negative control) or FAM3B siRNA for 48 h in DMEM medium containing 5.5 mM or 25 mM glucose. Cell proliferation was evaluated using the EdU incorporation (**A**,**B**) or direct cell counting assay (**C**). (**D**) Assessment of the cell cycle progression by flow cytometry. (**E**) Protein expression levels of key regulators involved in cell cycle progression. (**F**) Determination of VSMC migration by transwell chamber (top) and wound-healing (bottom) assays. (**G**,**H**) Quantification of the data from (**F**). (**I**) Protein expression levels of ICAM-1, VCAM-1, and MMPs. (**J**,**K**) Total activity of MMP-9 and MMP-2. ^**^ *P* < 0.01 *vs*. scra/5.5 mM glucose, ^*##*^ *P* < 0.01 *vs*. scra/25 mM glucose.

To confirm that FAM3B regulates VSMC proliferation and migration in a cell-autonomous manner, we knocked down FAM3B in high glucose-challenged cultured rat VSMCs. The knockdown efficiency was validated and shown in Fig. S6A,B. EdU incorporation and directly cell counting assays indicated that knockdown of FAM3B significantly inhibited high glucose-induced VSMC proliferation (Fig. 3A–C). However, the activity of Caspase-3 and Caspase-9 was not altered (Fig. S6C,D), suggesting that the suppression of VSMC proliferation by FAM3B silencing was not due to the possible occurrence of cell apoptosis. In addition, knockdown of FAM3B increased the percentage of cells quiescent at the G0/G1 phase and inhibited high glucose-induced S-phase re-entry (decreased from 11.6 ± 0.32% to 5.33 ± 0.96%) (Fig. 3D). At the molecular level, FAM3B knockdown reduced the protein expression levels of Cyclin E/D1, CDK2/6 and PCNA, whereas increased p27 protein expression (Figs 3E and S6E).

Similarly, FAM3B knockdown retarded high glucose-stimulated VSMC migration (Fig. 3F–H). The migration and adhesion-associated proteins also showed decreased expression levels in FAM3B-silenced VSMCs (Figs 3I and S6F). Specifically, total activity of MMP-9 and MMP-2 was reduced (Fig. 3J,K). Collectively, these results indicated that FAM3B mediates high glucose-induced VSMC proliferation, migration and adhesion.

### FAM3B accelerates proliferation and migration

Since FAM3B can be secreted into circulation, it is interesting to know that whether exogenous FAM3B is able to regulate VSMC behavior. VSMCs were treated with recombinant FAM3B protein for 48 h and we found their proliferation was accelerated (Fig. S7A,B for the EdU incorporation assay; Fig. S7C for the directly cell counting assay). Accordingly, the percentage of S-phase cells was increased (basal: 2.67 ± 0.19%; 0.4 nM FAM3B stimulation: 4.44 ± 0.29%; 4 nM FAM3B stimulation: 6.22 ± 0.19%), while G0/G1 phase cells were decreased (Fig. S7D). Western blot analysis indicated that exogenous FAM3B increased the protein expression levels of Cyclin E/D1, CDK2/6 and PCNA in a dose-dependent manner, but the expression of p27 was suppressed (Fig. S7E).

For the VSMC migration, addition of recombinant FAM3B protein also showed a stimulative function (Fig. S7F–H). Furthermore, the expression levels of migration and adhesion-associated proteins (Fig. S7I), as well as the total activity of MMP-9 and MMP-2 (Fig. S7J,K), were significantly increased by FAM3B protein.

**Figure 4:**
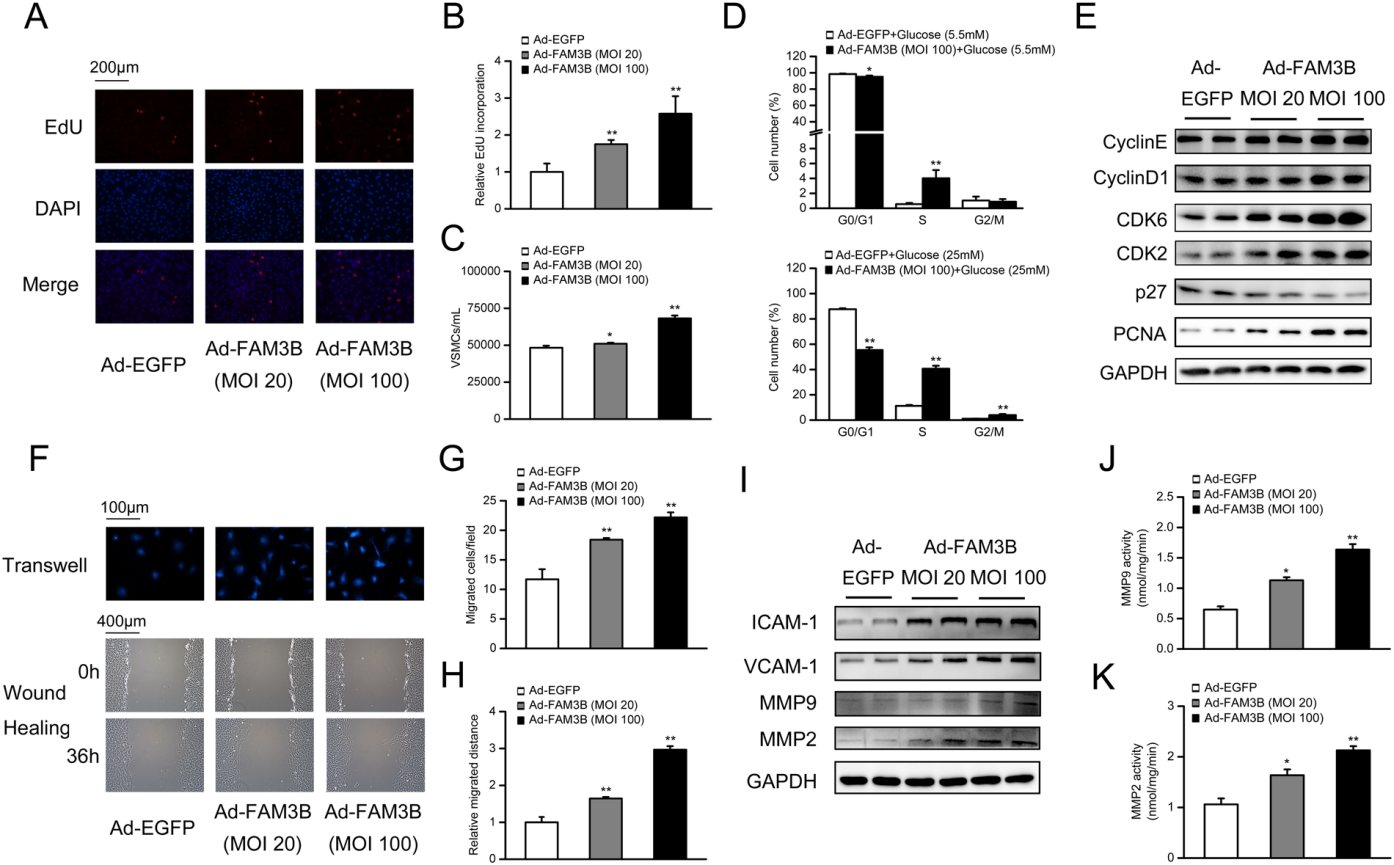
Overexpression of FAM3B accelerates VSMC proliferation and migration. VSMCs were infected with recombinant adenoviruses expressing either EGFP (as negative control) or FAM3B for 48 h in DMEM medium containing 5.5 mM glucose. Cell proliferation was evaluated using the EdU incorporation (**A**,**B**) or direct cell counting assay (**C**). ^*^ *P* < 0.05 and ^**^ *P* < 0.01 *vs*. Ad-EGFP group. (**D**) Assessment of the cell cycle progression by flow cytometry. VSMCs were similarly infected with adenoviruses in DMEM medium containing 5.5 mM or 25 mM glucose, respectively. ^*^ *P* < 0.05 and ^**^ *P* < 0.01 *vs*. Ad-EGFP/5.5 mM glucose; ^*##*^ *P* < 0.01 *vs*. Ad-EGFP/25 mM glucose. (**E**) Protein expression levels of key regulators involved in cell cycle progression. (**F**) Determination of VSMC migration by transwell chamber (top) and wound-healing (bottom) assays. (**G**,**H**) Quantification of the data from (**F**). ^**^ *P* < 0.01 *vs*. Ad-EGFP group. (**I**) Protein expression levels of ICAM-1, VCAM-1, and MMPs. (**J**,**K**) Total activity of MMP-9 and MMP-2. ^**^ *P* < 0.01 *vs*. Ad-EGFP group.

In the following study, we used gain-of-function strategy to show that FAM3B itself can sufficiently induce VSMC proliferation and migration. VSMCs were infected with adenoviruses expressing EGFP (as control) or FAM3B. 48 h after initial transduction, FAM3B expression was markedly increased by Ad-FAM3B (Fig. S8A,B). Interestingly, VSMC proliferation was accelerated by the forced expression of exogenous FAM3B in a dose-dependent manner (Fig. 4A,B for the EdU incorporation assay; Fig. 4C for the directly cell counting assay). In addition, FAM3B increased the percentage of S-phase cells under both low (increased from 0.56 ± 0.17% to 4.01 ± 1.11%) and high (increased from 11.26 ± 0.27 to 40.65 ± 2.67%) glucose conditions. Correspondingly, the cell numbers at the G0/G1 phase were decreased (Fig. 4D). At the molecular level, we found that the protein expression levels of Cyclin E/D1, CDK2/6 and PCNA were up-regulated by FAM3B overexpression in a dose-dependent manner (Fig. 4E, Fig. S8C), whereas suppressed the expression of p27.

Similar to the proliferation results, FAM3B overexpression promoted VSMC migration (Fig. 4F–H). Moreover, as is shown in Figs 4I and S8D, FAM3B significantly increased the migration/adhesion associated protein expression levels in a dose-dependent manner. Besides, the total activity of MMP-9 and MMP-2 was increased when FAM3B was overexpressed (Fig. 4J,K).

### FAM3B inhibits miR-322-5p expression

**Figure 5:**
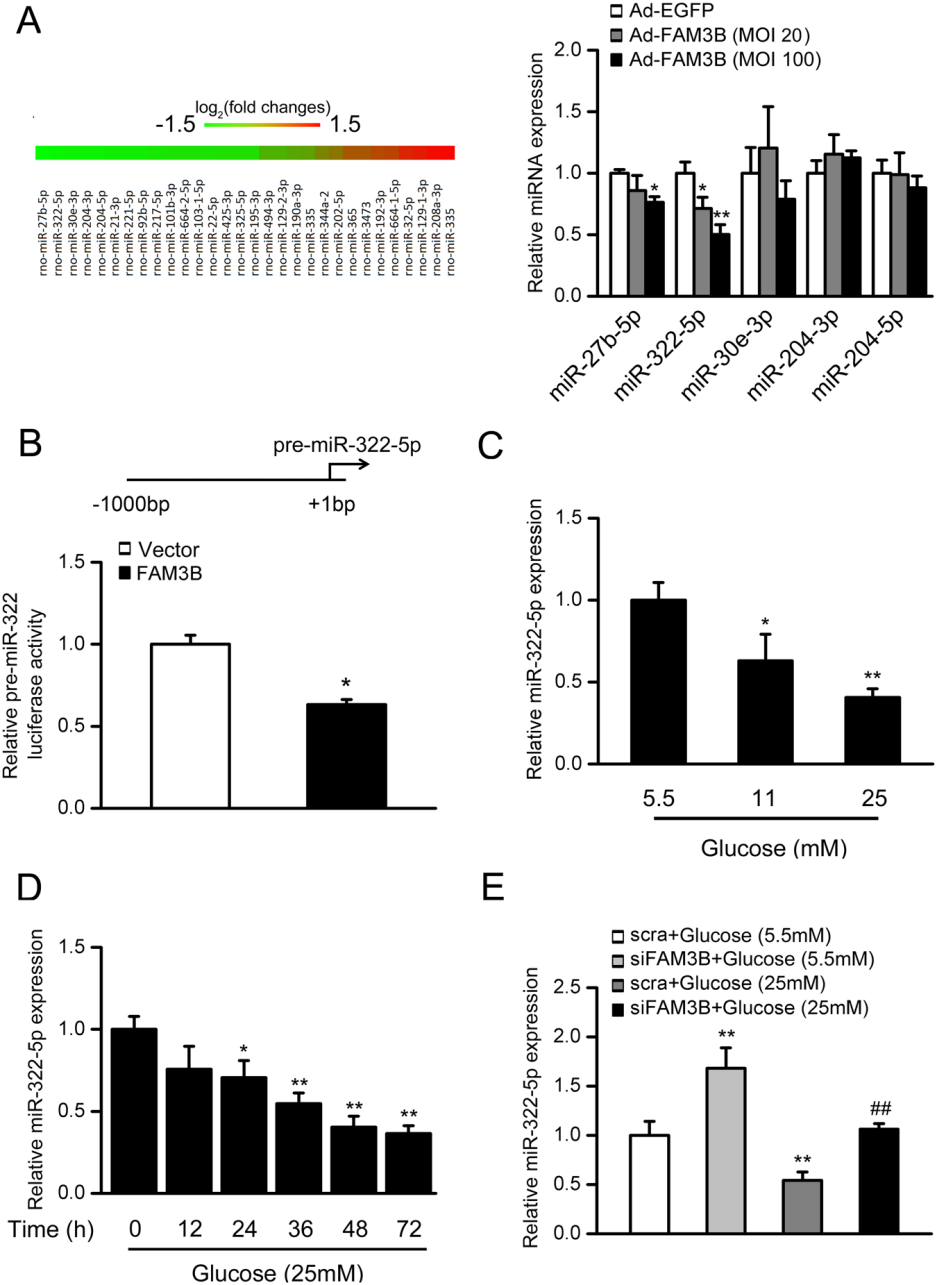
Overexpression of FAM3B inhibits miR-322-5p expression. (**A**) Microarray analysis of miRNA expression in VSMCs infected with adenoviruses expressing either EGFP or FAM3B (left). RT-qPCR validation of miRNAs potentially downregulated by FAM3B (right). ^*^ *P* < 0.05 and ^**^ *P* < 0.01 *vs*. Ad-EGFP. (**B**) Analysis of the transcriptional activity of pre-miR-322 promoter by reporter gene assays. ^*^ *P* < 0.05 *vs*. vehicle. (**C**) RT-qPCR analysis of miR-322-5p expression in VSMCs stimulated with various concentrations of glucose for 36 h. ^*^ *P* < 0.05 and ^**^ *P* < 0.01 *vs*. 5.5 mM glucose. (**D**) RT-qPCR analysis of miR-322-5p expression in VSMCs stimulated with 25 mM glucose for the indicated time-points. ^*^ *P* < 0.05 and ^**^ *P* < 0.01 *vs*. 0 h. (**E**) RT-qPCR analysis of miR-322-5p expression in VSMCs with FAM3B knockdown. ^**^ *P* < 0.01 *vs*. scra/5.5 mM glucose, ^*##*^ *P* < 0.01 *vs*. scra/25 mM glucose.

MiRNAs have been shown to play a pivotal role in the regulation of VSMC physiology including cellular proliferation and migration^27,28^. To pinpoint whether miRNAs were involved in FAM3B-induced activation of VSMCs, we performed miRNA microarray analysis. As shown in Fig. 5A, microarray profile in FAM3B-overexpressed VSMCs identified a bunch of miRNAs potentially serving as downstream targets of FAM3B. Among which, the expression levels of miR-27b-5p, miR-322-5p, miR-30e-5p, miR-204-3p and miR-204-5p were significantly decreased. To confirm these results, an independent RT-qPCR assay was performed. The results indicated that the expression of miR-322-5p showed a maximum decrease in response to the overexpression of FAM3B in VSMCs, and this regulation is in a dose-dependent manner (Fig. 5A). Additionally, reporter gene assays demonstrated that FAM3B repressed the transcriptional activity of pre-miR-322 promoter by 37% (Fig. 5B). Physiologically, glucose has been shown to inhibit miR-322 expression in C17.2 neural cells^29^, we therefore investigated whether such an inhibition exists in VSMCs. Indeed, RT-qPCR analysis revealed that glucose decreased miR-322-5p expression in a dose- and time-dependent manner (Fig. 5C,D). To investigate the possible mediatory role of FAM3B in the downregulation of miR-322-5p by glucose, we knocked down FAM3B in VSMCs and found that the inhibitory effect of glucose on miR-322-5p expression was significantly alleviated (Fig. 5E).

### MiR-322-5p blocks FAM3B-induced proliferation and migration

**Figure 6:**
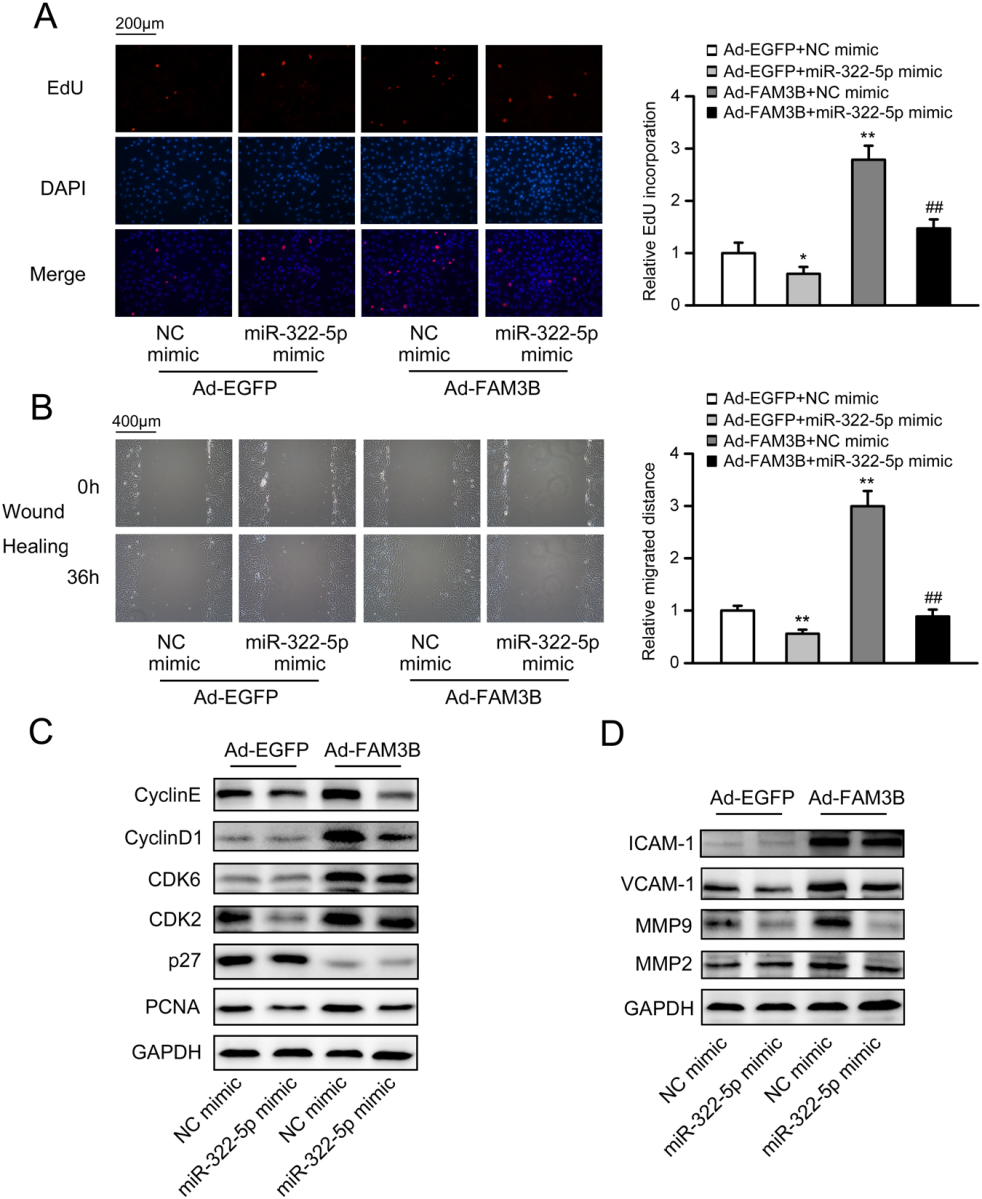
MiR-322-5p blocks FAM3B-induced VSMC proliferation and migration. VSMCs were infected with adenoviruses expressing EGFP or FAM3B for 36 h. Prior to the virus infection, negative control (NC) mimic or miR-322-5p mimic was transfected into VSMCs for 12 h when indicated. (**A**) Determination of cell proliferation by the EdU incorporation assay. (**B**) Determination of cell migration by the wound-healing assay. ^*^ *P* < 0.05 and ^**^ *P* < 0.01 *vs*. Ad-EGFP/NC mimic, ^*##*^ *P* < 0.01 *vs*. Ad-FAM3B/NC mimic. (**C**,**D**) Protein expression levels of key regulators involved in the cell cycle progression (**C**), cell migration and adhesion (**D**).

Since miR-322 has been reported to possess anti-proliferative and anti-migratory properties, and promote VSMC differentiation without affecting apoptosis^30,31^, we hypothesized that miR-322-5p could be a downstream mediator in FAM3B-induced VSMC activation. To test our hypothesis, miR-322-5p mimic was transfected into VSMCs (the transfection efficiency was validated and shown in Fig. S9A) and the cell proliferation and migration rates were assessed. Results from the EdU incorporation assay revealed that exogenous miR-322-5p expression inhibited both basal and FAM3B-induced VSMC proliferation (Fig. 6A). FAM3B-induced VSMC migration was also antagonized by miR-322-5p (Fig. 6B). At the molecular level, miR-322-5p mimic decreased FAM3B-induced accumulation of cell cycle-associated (Cyclin D1/E, CDK2, PCNA) and adhesion-related (VCAM-1 and MMP-9) proteins, whereas other regulators such as CDK6, p27, ICAM-1 and MMP-2 remained unchanged (Fig. 6C,D), suggesting the specificity of this regulation. Quantitative data of these results were presented in Fig. S9B,C.

## Discussion

In the present study, we demonstrated a novel role of FAM3B in regulating VSMC proliferation and migration in response to hyperglycemia. FAM3B expression was induced in the VSMC layer of the thoracic aorta of hyperglycemic rats and in glucose-challenged cultured VSMCs. FAM3B over-expression greatly accelerated basal VSMC proliferation and migration rates, while FAM3B knockdown inhibited glucose-induced VSMC abnormal activation. We also revealed that such FAM3B’s effects were dependent, at least in part, on inhibition of miR-322-5p.

FAM3B is a member of the FAM3 family of cytokine molecules initially described in 2002. During the last decade, the physiological roles of FAM3B have been extensively studied. FAM3B is highly expressed in the endocrine pancreas and is secreted from both pancreatic α- and β-cells^32^. *In vitro*, recombinant FAM3B pretreatment or viral-mediated FAM3B overexpression promotes apoptosis of islet β-cells and impairs glucose-stimulated insulin secretion^18,23^. Chronic hyperglycemia activates FAM3B expression and couples the secretion of FAM3B and insulin under conditions of insulin resistance^33^. Unexpectedly, liver is a major target tissue for FAM3B. FAM3B binds to the liver cell membrane and induces insulin resistance, leading to increased gluconeogenesis^24,25,34^. FAM3B is also expressed within liver and promotes hepatic lipogenesis^33^. Therefore, FAM3B plays an important role in the progression of type 2 diabetes by negatively regulating islet function and insulin sensitivity in the liver. It should be noted that diabetes is associated to an increased risk of cardiovascular diseases^35^. Hyperglycemia is an important factor to trigger cardiovascular damage by working through different mechanisms, such as promotion of VSMC proliferation and migration^9^. However, the molecules directing linking glucose and VSMC physiology remain largely unknown. In this study, we showed that FAM3B responds to glucose sensitively and in turn promotes VSMC proliferation and migration. These findings highlight the importance of FAM3B not only in the pathogenesis of diabetes, but also in the development of diabetes-related cardiovascular complications. In addition, FAM3B is a secretory protein, containing a typical secretory signal peptide. Thus, hyperFAM3Bemia induced by hyperglycemia and hyperlipidemia seen in type 2 diabetes would build up a critical crosstalk among various metabolic tissues, including islets, liver and VSMCs, leading to a systemic dysfunction in the control of metabolic homeostasis. In this sense, inhibition of FAM3B deleterious actions may be consistently beneficial for metabolic tissues and provide a therapeutic option to prevent the further deterioration of glycemic control and cardiovascular functions, achieving the effects of killing two birds with one stone.

At the molecular level, hyperglycemia induces VSMC abnormalities through various metabolic pathways, such as activation of PKC and generation of advanced glycation end products^36,37^. All these pathways, in association to hyperglycemia-induced mitochondrial dysfunction and ER stress, promote ROS accumulation in VSMCs^38^. ROS can directly damage lipids, proteins or DNA and activate intracellular signaling pathways, such as MAPKs and redox sensitive transcriptional factors, leading to modulation of the expressions of genes involved in inflammation including intracellular adhesion molecules^39^. On the other hand, high glucose enhances the expression and activity of MMP-2 and MMP-9, which are the principle enzymes in extracellular matrix degradation essential for the VSMC invasion^40^. All these pathways activated by hyperglycemia contribute to the increased VSMC proliferation and migration. In the present study, we found that a bunch of miRNAs potentially regulated by FAM3B, among which, miR-322-5p showed a maximum decreased expression level and such a decrease was in a dose-dependent manner. More importantly, overexpression of miR-322-5p through miR-322-5p mimic attenuated the stimulative effects of FAM3B on VSMCs. These data demonstrate that the regulation of miRNA by FAM3B has a high specificity and miR-322-5p mediates FAM3B-induced abnormal activation of VSMCs.

In addition to pancreas, FAM3B is ubiquitously expressed in most human normal tissues and cancers^41^. Interestingly, the roles of FAM3B in the regulation of tumor cell behaviors have been preliminarily described. Mou *et al*. showed that inhibition of FAM3B expression induced apoptosis in HCT8 human colon cancer cells through p53-dependent pathway, suggesting that FAM3B is required for the cell survival^41^. In contrast, stable overexpression of FAM3B-258, a non-secretory form of FAM3B, in colon cancer cells led to increased expression levels of Slug and Cdc42 and promoted cell migration and invasion *in vitro* and metastasis in nude mice^42^. On the other hand, miR-424, the human ortholog of miR-322, is significantly decreased in human cancer cells, which functions as a promising tumor suppressor^43^. Actually, atherosclerosis and cancer share common mechanisms, which is responsible for the higher incidence of thromboembolic events in cancer patients, the occurrence of similar laboratory findings and the effects of many drugs on the course of these two diseases^44,45^. For example, atherosclerosis may develop when an injury or infection mutates or transforms a single VSMC in the progenitor of a proliferative clone, similar to the most widely accepted carcinogenesis theory^44^. Cell proliferation regulatory pathways have also been associated with plaque progression, stenosis, and restenosis after angioplasty and with cancer progression^46–48^. In addition, alterations in adhesion molecules contribute to the plaque formation and thrombosis and to tumor invasion and metastasis^49,50^. Thus, the regulation of cell proliferation and migration by FAM3B could be universally occurring in VSMCs and tumor cells. Targeting FAM3B will therefore serve as a novel therapeutic strategy against both atherosclerosis and cancer.

**Figure 7:**
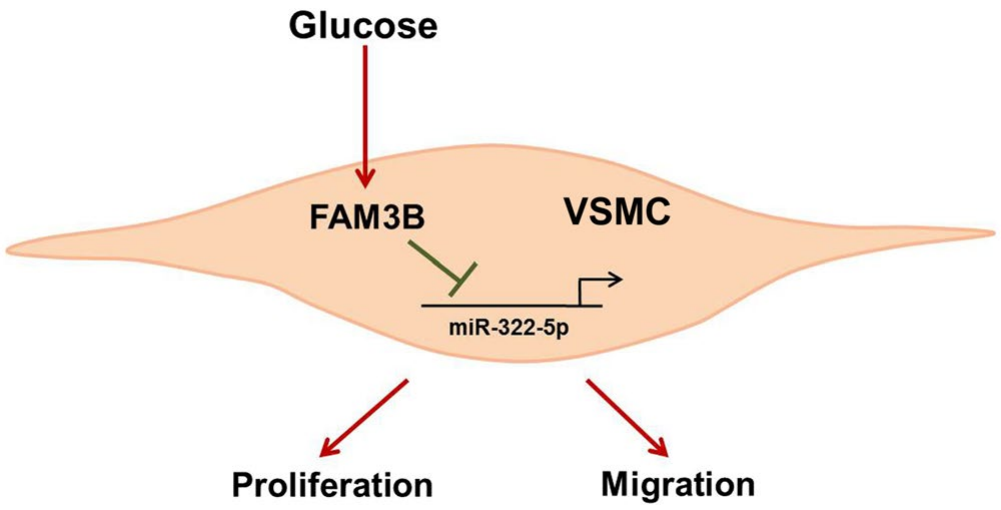
FAM3B mediates high glucose-induced VSMC proliferation and migration via inhibition of miR-322-5p.

In conclusion, our results provide evidences showing that FAM3B is a critical chemokine required for high glucose-induced VSMC proliferation and migration. The effects of FAM3B are achieved through inhibition of miR-322-5p (Fig. 7). Given that the increasing incidence of diabetes and subsequent vascular diseases is currently a major public health problem in industrialized countries, inhibition of FAM3B expression in VSMCs may be beneficial for the prevention and therapy of diabetes-associated cardiovascular complications.

## Materials and Methods

### Animals

All animal procedures in this investigation were performed according to the Guide for the Care and Use of Laboratory Animals published by the US National Institutes of Health (NIH publication No. 85–23, revised 1996) and the approved regulations set by the Laboratory Animal Care Committee at China Pharmaceutical University. Male Sprague–Dawley (SD) rats, weighing 180–200 g, were maintained in a 12 h light/12 h dark cycle (light: dark 12:12) in a temperature- and humidity-controlled environment. To induce hyperglycemia, overnight-fasted rats were single intraperitoneally (i.p.) injected with streptozotocin (STZ, Sigma-Aldrich, St. Louis, MO, USA.) at the dose of 55 mg/kg body mass. Control rats were received with equal amount of vehicle (sodium citrate solution). Four and six weeks after STZ injection, rats were killed by cervical dislocation. To detect the expression of FAM3B in the VSMC layer of thoracic aorta of hyperglycemic mice, 7-week-old diabetic *db*/*db* mice on a C57BKS background were purchased from the Model Animal Research Center of Nanjing University (Nanjing, Jiangsu, China). After acclimatization for one week, mice were killed to collect thoracic aorta samples.

### Morphometric analysis & Immunohistochemistry

Rat thoracic aortas were isolated, fixed in 4% paraformaldehyde solution for 24 h *in situ*, possessed for paraffin embedding, and cut into 4 µm transverse sections. The tissue sections were either subjected to Masson-Goldner trichrome staining for the detection of collagen fibers and muscle fibers. Six cross-sectional areas of three blood vessel layers (the intima, media and adventitia) were randomly selected and measured with a DP70 digital camera connected to a microscope (Olympus BX41).

### Immunofluorescence assay

Mouse thoracic aortas were isolated and frozen sections were blocked in 5% goat serum 37 °C for 1 h, and then incubated at 4 °C overnight with the antibody against α-SMA (Cat. No. 14395; 1:100 dilution, Proteintech), FAM3B (Cat. No. sc-83250; 1:50 dilution, Santa Cruz) or SM-MHC (Cat. No. sc-6956; 1:50 dilution). To detect the co-localization of SM-MHC and FAM3B (Fig. 1B), tissue sections were incubated with secondary antibodies conjugated to Alexa Fluor 488 (Cat. No. 4408; 1:500 dilution, Cell Signaling Technology) and Alexa Fluor 555 (Cat. No. 4413; 1:500 dilution, Cell Signaling Technology) for 30 min at room temperature. For Fig. S1A, secondary antibodies conjugated to Alexa Fluor 488 (Cat. No. 4412; 1:500 dilution, Cell Signaling Technology) and Alexa Fluor 555 (Cat. No. 4413; 1:500 dilution, Cell Signaling Technology) were instead used. Nuclei were identified with DAPI. Non-immune IgG was used as a negative control. The sections were photographed with a DP70 digital camera connected to a microscope (Olympus BX41).

### Cell culture

Primary VSMCs were isolated from the thoracic aortas of normal rats (3–4 week-old) and STZ-induced hyperglycemic rats. All VSMCs were characterized morphologically and immunohistochemically as described previously^51^. Cells at passages 4 to 6 were applied in all experiments. Growth-arrested VSMCs were incubated in DMEM containing different concentrations of glucose. Mannitol was used to balance the osmotic pressure. For free fatty acids (FFAs) and insulin stimulation, VSMCs were treated with either 0.4 mM FFAs (an equal molar mixture of oleic acid and palmitic acid) or 100 nM insulin for 24 h. The concentrations and the treatment time of FFAs or insulin were selected based on previous studies showing their positive effects to activate VSMCs^52,53^. To investigate the regulation of VSMC behavior by exogenous FAM3B, VSMCs were treated with recombinant FAM3B protein (0.4 nM and 4 nM, Cloud-clone Corp, Wuhan, Hubei, China) for 48 h in DMEM medium containing 5.5 mM glucose^23^.

### RT-qPCR

Total RNA from VSMCs were extracted using Trizol reagent (Invitrogen, Carlsbad, CA, USA). For mRNA detection, 2 μg of total RNA was reverse-transcribed into complementary DNA. Real-time PCR amplification was performed using SYBR premix Ex Taq (Vazyme, Nanjing, Jiangsu, China) and the LightCycler® 96 System (Roche, Basal, Switzerland). 18S ribosomal RNA served as an internal control to normalize the expression levels of mRNAs. Primer sequences were: 5′-TAGAAGCCCTTGGAAGCAAA-3′ (F) and 5′-CCTTCGATCTGGATCTCTGC-3′ (R) for *Fam3b*; 5′-AAACGGCTACCACATCCAAG-3′ (F) and 5′-CCTCCAATGGATCCTCGTTA-3′ (R) for *18S rRNA*. For miRNA quantification, miR-322-5p bulge-loop was reverse transcribed with the PrimeScript RT reagent kit (Takara, Tokyo, Japan) and quantified by qPCR. MiRNA expression was normalized to snRNA U6 to get the relative abundance.

### Western blot

Cultured VSMCs were lysed in RIPA buffer. The protein concentration was quantified with a BCA protein quantification kit (KeyGen, Nanjing, Jiangsu, China). Equal amounts of protein were loaded and separated by 10% SDS-PAGE and then transferred onto PVDF membranes (Millipore, Bedford, MA, USA). The membranes were incubated overnight with appropriate primary antibodies. HRP-conjugated secondary antibodies were then applied to bind and visualize the primary antibodies. Quantitative analysis was performed by NIH Image J 1.32j software. For the antibody information, antibodies against Cyclin E (Cat. No. 4129; 1:1000 dilution), PCNA (Cat. No. 2586; 1:1000 dilution), CDK2 (Cat. No. 2546; 1:1000 dilution), Cyclin D1 (Cat. No. 2922; 1:1000 dilution), CDK6 (Cat. No. 3136; 1:1000 dilution), and p27 (Cat. No. 3686; 1:1000 dilution) were purchased from Cell Signaling Technology (Danvers, MA, USA). Antibodies against ICAM-1 (Cat. No. sc-1511; 1:500 dilution), VCAM-1 (Cat. No. sc-1504; 1:500 dilution), MMP-2 (Cat. No. sc-10736; 1:500 dilution), MMP-9 (Cat. No. sc-6840; 1:500 dilution), SM-MHC (Cat. No. sc-6956; 1:500 dilution), FAM3B (Cat. No. sc-83250; 1:500 dilution), and GAPDH (Cat. No. sc-47724; 1:5000 dilution) were purchased from Santa Cruz (Dallas, TX, USA). Antibodies against α-SMA (Cat. No. 14395; 1:1000 dilution), SM22α (Cat. No. 10493; 1:1000 dilution), SM-calponin (Cat. No. 13938; 1:1000 dilution) were purchased from Proteintech (Chicago, IL, USA).

### Protein half-life analysis

The half-life of FAM3B protein was measured by the CHX chase assay. VSMCs were incubated in DMEM medium containing 5.5 or 25 mM glucose in presence of 10 μg/mL CHX (Sigma). Cells were harvested at the indicated time-points and subjected to Western blot analysis.

### Protein stability assay

The protein stability of FAM3B protein was measured by co-immunoprecipitation (Co-IP) assay targeting FAM3B ubiquitination. VSMCs were incubated in DMEM medium containing 5.5 or 25 mM glucose for 34 h, followed by the treatment with either 10 μM MG132 (Sigma) or DMSO (vehicle) for 2 h. Cells were lysed and lysates were precipitated with anti-FAM3B antibody and pre-cleared protein A/G PLUS-Agarose beads (Roche, Basal, Switzerland). Ubiquitinated proteins within the IP were detected by Western blot using a monoclonal anti-ubiquitin antibody ((Cat. No. sc-8017; 1:500 dilution, Santa Cruz).

### Adenovirus infection and siRNA transfection for FAM3B

The adenoviruses expressing EGFP and FAM3B were constructed by Yingrun Corporation (Changsha, Hunan, China). To knockdown FAM3B expression in VSMCs, three sets of stealth siRNAs for FAM3B were designed and synthesized by Invitrogen. To improve gene silencing efficiency, a siRNA cocktail comprising these three sets of siRNA oligonucleotides (an equal molar mixture) was used. The sequences of these siRNA oligonucleotides were: #1: sense, 5′-GAGCCAAUGACUAAGUUUAUUCAGA-3′, anti-sense, 5′-UCUGAAUAAACUUAGUCAUUGGCUC-3′; #2: sense, 5′-UCCCUUCGAAAUCCCUGCUGUUCAU-3′, anti-sense, 5′-AUGAACAGCAGGGAUUUCGAAGGGA-3′; #3: sense, 5′-ACUUUGACAUGUACGAAGGUGAUAA-3′, anti-sense, 5′-UUAUCACCUUCGUACAUGUCAAAGU-3′.

### Proliferation assay

Cell proliferation was analyzed by using EdU incorporation and cell counting assays. For the EdU incorporation assay, VSMCs were incubated with 50 μM EdU for 12 h and fixed with 4% paraformaldehyde. EdU incorporation was determined by using anti-EdU antibody (Promega, Madison, WI, USA). Cells were double stained with DAPI (Sigma, St. Louis, MO, USA). Signals were visualized by a fluorescence microscope (Nikon, Tokyo, Japan, Ti-S 533665), and the average ratios between EdU-positive (pink) and total DAPI-stained nuclei (blue) were calculated for statistic analyses. For the cell counting, 5 × 10^4^ VSMCs were seeded into each well of 6-well plates. After treatments, cells were re-suspended with 0.05% trypsin and 0.02% EDTA and counted by a hemocytometer.

### Flow cytometry

VSMCs were fixed with 70% ethanol overnight, washed twice with PBS, and stained with a 50 μg/mL propidium iodide (PI) solution containing 0.1% Triton X-100, 0.1 mM EDTA, and 50 μg/mL RNase A. 1 h later, the fluorescence of PI-DNA complex in nuclei was measured using a FACS Calibur instrument (Becton-Dickinson, Franklin, NJ, USA). The rates of the G0/G1, S, and G2/M phases of the cell cycle were determined using Modfit LT software.

### Migration assay

Cell migration was analyzed by using wound-healing and transwell chamber assays. For the wound-healing assay, VSMCs were seeded in 6-well plates (1.5 × 10^5^ cells per well) and grew to confluence. 24 h after serum deprivation, the cells were subjected to different treatments as indicated. To eliminate the possibility that the cell migration is dependent on proliferation, we next treated the cells with mitomycin C (20 μM), a potent DNA crosslinker, for 2 h and mounted cells to a reusable template to create a standard wound (<3 mm) (This time point was set as 0 h). Wound closure rates were followed with a reference point in the field of the wound at the bottom of the plate by direct microscopic visualization. The procedure permitted photographing the identical spot each time. The remaining cell-free area was determined via microphotography and performed immediately after 36 h injury. For the transwell chamber assay, a 24-well modified Boyden chamber containing fibronectin-coated polycarbonate membranes (8 μm pore-size, BD Bioscience, Franklin, NJ, USA) was used. Briefly, the lower wells of the chamber were filled with phenol red-free DMEM. The filters were coated with 50 mg/mL fibronectin and fixed atop the bottom wells. 1 × 10^5^ per well VSMCs were allowed to migrate for 6 h and non-migrated cells were removed from the upper side of the membrane with cotton swabs. Cells on the lower side of the membrane were stained with Hoechst 33342, and then counted in five randomly selected squares per well with a fluorescence microscope (Nikon, Tokyo, Japan, Ti-S 533665). Data was presented as numbers of migrated cells per field.

### Total MMP activity

The total activity of MMP-2 and MMP-9 was determined using a GenMed MMP Fluorimetric Assay Kit (GenMed Scientifics Inc., Shanghai, China) according to the manufacturer’s instructions.

### Caspase-3 and Caspase-9 activity

The activity of Caspase-3 and Caspase-9 was evaluated by using commercial kits (Beyotime Biotechnology, Nantong, Jiangsu, China) according to the manufacturer’s protocols.

### MiRNA microarray analysis

VSMCs were transduced with adenoviruses expressing EGFP and FAM3B for 48 h in DMEM medium containing 5.5 mM glucose. Total RNA was isolated and subjected to the Affymetrix GeneChip miRNA 4.0 array (Gene Tech, Shanghai, China).

### Luciferase reporter assay

A putative pre-miR-322 promoter was constructed by PCR amplification as described previously^54^. The PCR product was inserted between the Xho l and Mlu I restriction enzyme cutting sites, immediately downstream of the luciferase gene in the pRL-TK Vector. VSMCs were transfected with 300 ng of reporter plasmids and 1 μg of expression constructs for FAM3B. Equal amounts of DNA were used for all transfection combinations by adding the appropriate vector DNA. Relative luciferase activities were determined 48 h following transfection using the Dual Luciferase system (Promega, Madison, WI, USA). The transfection experiments were performed in triplicate.

### MiRNA mimic

Chemically modified double-stranded RNAs were designed and synthesized by Ribobio (Guangzhou, Guangdong, China) to mimic the endogenous miR-322-5p. These RNA mimics were transfected into VSMCs using Lipofectamine 3000 (Invitrogen) according to the manufacturer’s instructions.

### Data analysis

Statistical analysis was performed by using the Origin 8 software (version 8.6, OriginLab Corporation, USA). Groups of data were presented as mean ± SD (standard deviation). One-way ANOVA followed by Fisher’s LSD post-hoc test were performed to analyze the data. A value of *P* < 0.05 was considered as statistically significant.

## Electronic supplementary material

Supplementary figure
