# Supplementary material for: FAM3B mediates high glucose-induced vascular smooth muscle cell proliferation and migration via inhibition of miR-322-5p
Source: Sci Rep. 2017 May 23;7:2298. doi: 10.1038/s41598-017-02683-3 (PMC5442163; doi:10.1038/s41598-017-02683-3)
Supplement: Supplementary file 1 — Supplementary figure [file 41598_2017_2683_MOESM1_ESM.pdf]

# FAM3B mediates high glucose-induced vascular smooth muscle cell proliferation and migration via inhibition of miR-322-5p

Wenxiang Zhang[1,2], Siyu Chen[1,3], Zhao Zhang[4], Chen Wang[1,2*] and Chang Liu[1,2,4*]

[1] School of Life Sciences, China Pharmaceutical University, Nanjing, Jiangsu, China.

[2] Jiangsu Key Laboratory of Drug Discovery for Metabolic Disease and State Key Laboratory of Natural Medicines, China Pharmaceutical University, Nanjing, Jiangsu, China. [3] School of Basic Medical Sciences and Jiangsu Key Laboratory of Human Functional Genomics, Nanjing Medical University, Nanjing, Jiangsu, China. [4] Jiangsu Key Laboratory for Molecular and Medical Biotechnology and College of Life Sciences, Nanjing Normal University, Nanjing, Jiangsu, China.

* Correspondence and requests for materials should be addressed to C.W. (email: cwang1971@cpu.edu.cn) or C.L. (email: changliu@njnu.edu.cn)

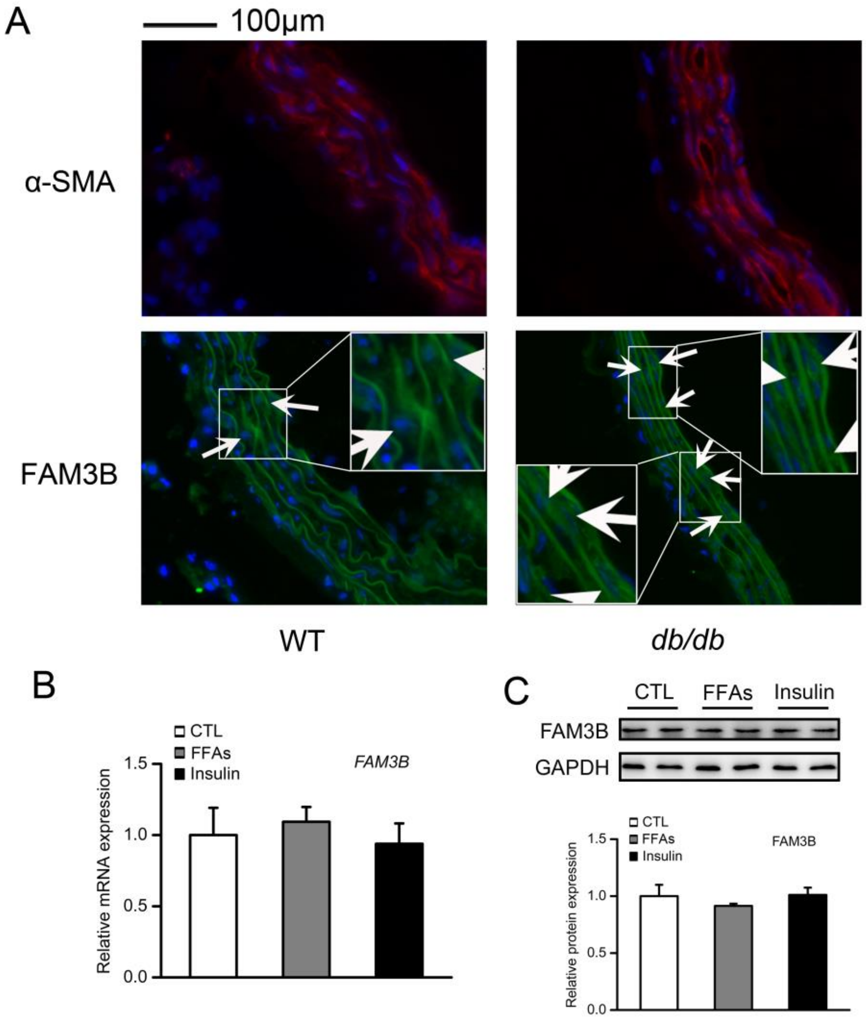


**Figure S1. Glucose, but not free fatty acids (FFAs) and insulin, induces FAM3B expression in VSMCs.** (A) Immunofluorescence assay of FAM3B protein expression in the VSMC layer of thoracic aortas isolated from wild type (WT) and diabetic *db/db* mice. Red: α-SMA; Green: FAM3B; Blue: DAPI. (B, C) Cultured rat VSMCs were stimulated with either 0.4 mM FFA (an equal molar mixture of oleic acid and palmitic acid) or 100 nM insulin for 24 h. (B) RT-qPCR and (C) Western blotting analyses of FAM3B expression levels.

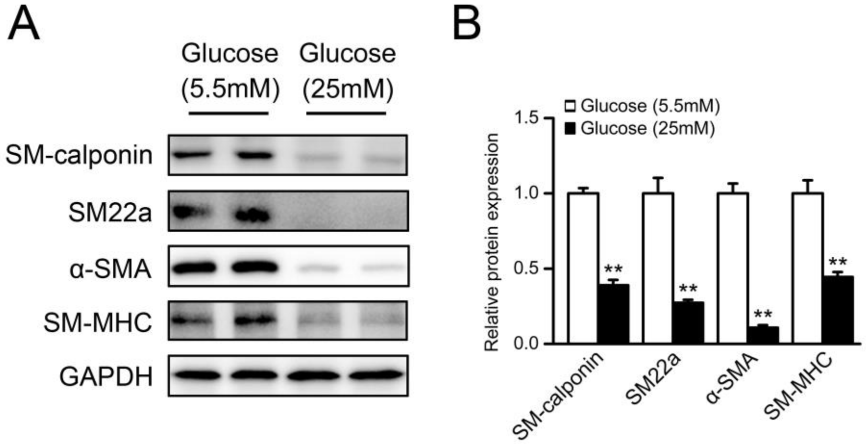


**Figure S2. Glucose inhibited protein expression levels of contractile marker genes in VSMCs.** Cultured rat VSMCs were stimulated with 25 mM glucose for 36 h. (A) Western blotting analysis for the protein expression of SM-MHC, SMA, SM22a and SM-calponin. (B) Quantification for the immunoblots shown in Fig. S2A. $^{**}P<0.01$ *vs.* 5.5 mM glucose.

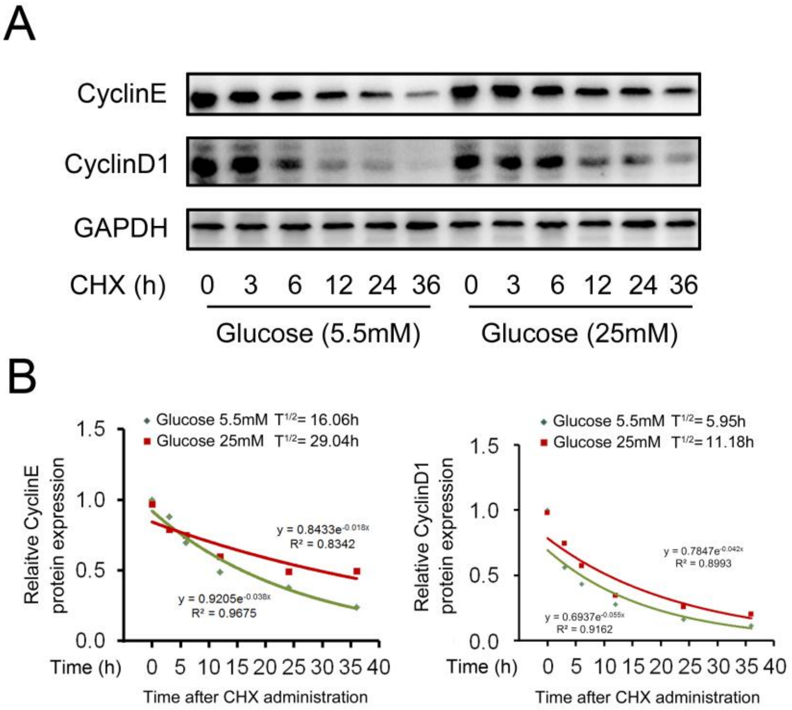


**Figure S3. Glucose stabilized Cyclin E and Cyclin D1 in VSMCs.** Cultured rat VSMCs were stimulated with 25 mM glucose for the indicated time-points. (A) Western blotting analysis for the protein expression of Cyclin E and Cyclin D1. (B) Quantification of the half-life times of cyclin E and cyclin D1 by a CHX chase experiment.

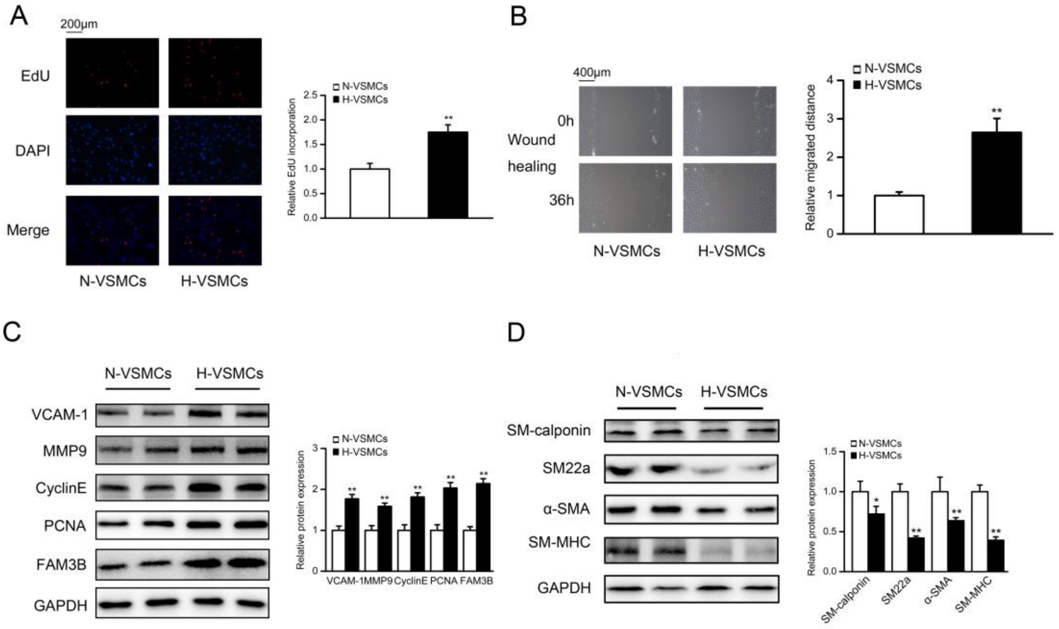


**Figure S4. VSMCs isolated from hyperglycemic rats exhibit accelerated proliferation and migration.** VSMCs were isolated from the thoracic aortas of rats 4 weeks after STZ injection and cultured in DMEM containing 25 mM glucose. (A) Determination of cell proliferation by the EdU incorporation assay. (B) Determination of cell migration by the wound-healing assay. (C) Protein expression levels of key regulators involved in the cell proliferation, migration and adhesion. (D) Protein expression levels of contractile marker genes. Data are represented as mean ± SD, the same below. $^{**}P$<0.01 *vs.* N-VSMCs.

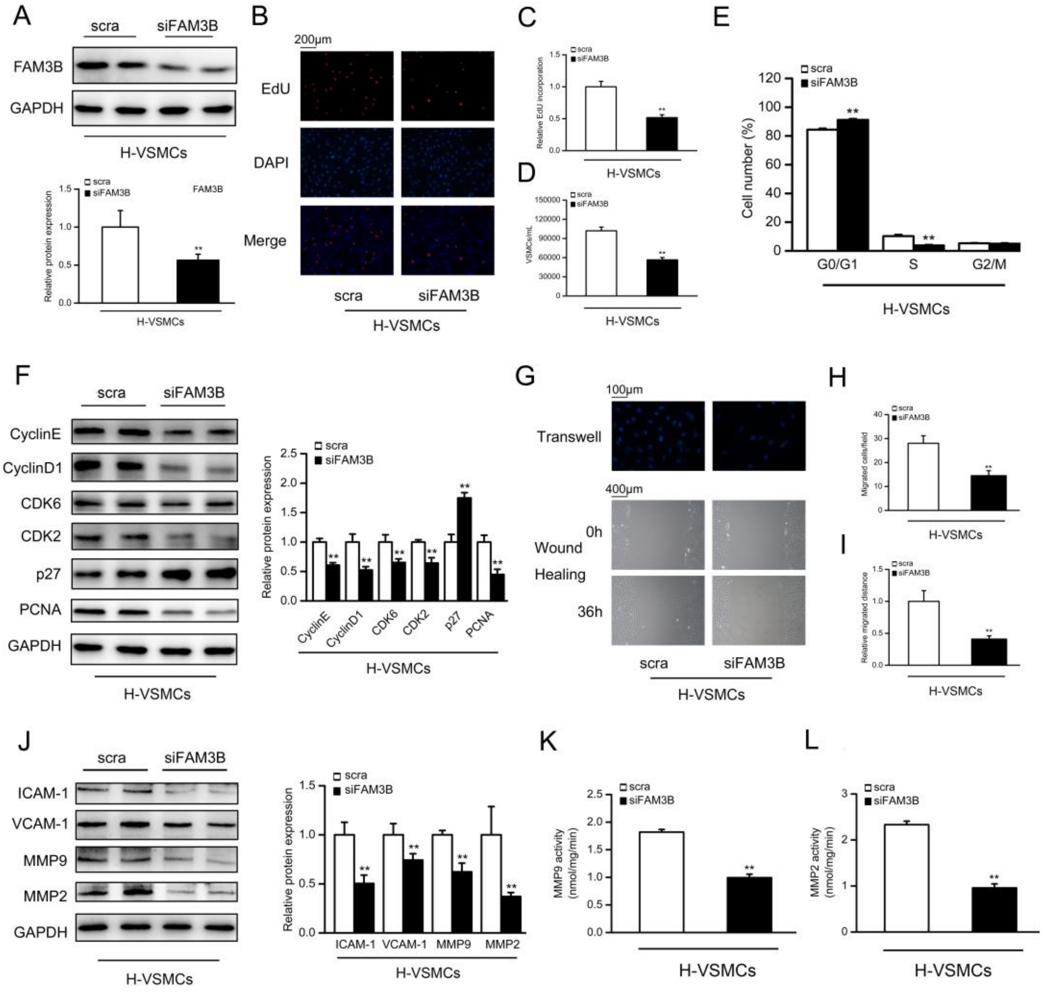

**Figure S5. Knockdown of FAM3B inhibits proliferation and migration of VSMCs isolated from hyperglycemic rats.** VSMCs isolated from STZ-induced hyperglycemic rats were transfected with either scramble (scra) siRNA (as negative control) or FAM3B siRNA for 48 h in DMEM medium containing 25 mM glucose. (A) The efficiency of FAM3B knockdown by siRNA transfection in H-VSMCs. **P<0.01 vs. scra. (B-D) Cell proliferation rate was evaluated by using EdU incorporation (B, C) or direct cell counting assays (D). (E) Assessment of the cell cycle progression by FACS. (F) Protein expression levels of key regulators involved in cell cycle progression. A representative image was shown from three separate experiments. (G) Determination of VSMC migration by transwell chamber (top) and wound-healing (bottom) assays. (H, I) Quantification of the data from (G). (J) Protein expression levels of ICAM-1, VCAM-1, and MMPs. (K, L) Total activity of MMP-9 and MMP-2. $^{**}P<0.01$ *vs.* scra.

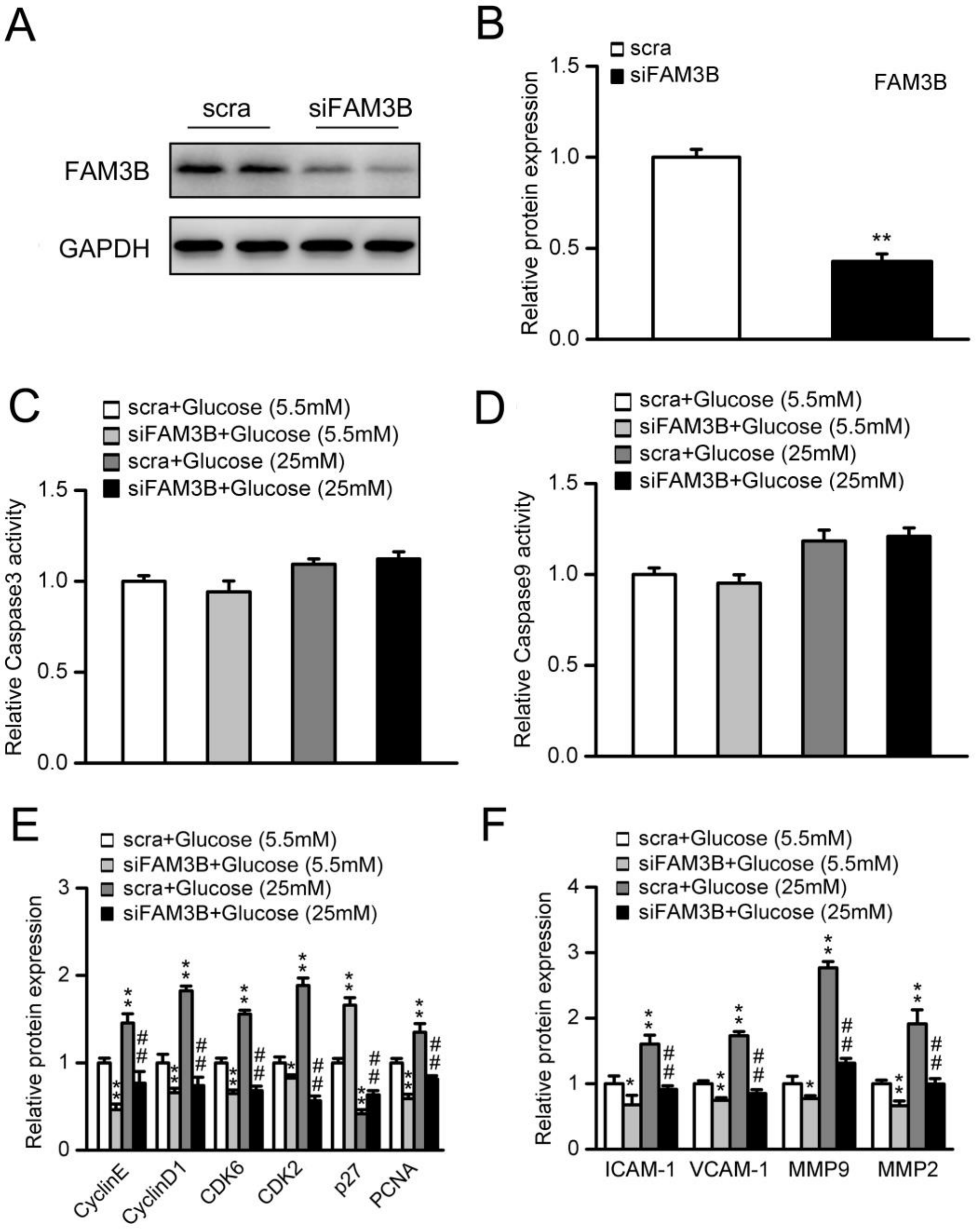


**Figure S6. Knockdown of FAM3B inhibits high glucose-induced VSMC proliferation and migration.** (A, B) The efficiency of FAM3B knockdown by siRNA transfection in high glucose-challenged VSMCs. ***P*<0.01 vs. scra. (C, D) FAM3B knockdown does not affect Caspase-3 (C) and Caspase-9 (D) activity. (E) Quantification for the immunoblots shown in Fig. 3E. (F) Quantification for the immunoblots shown in Fig. 3I. $^{*}$*P*<0.05 and $^{**}$*P*<0.01 *vs.* scra/5.5 mM glucose, $^{\#\#}$*P*<0.01 *vs.* scra/25 mM glucose.

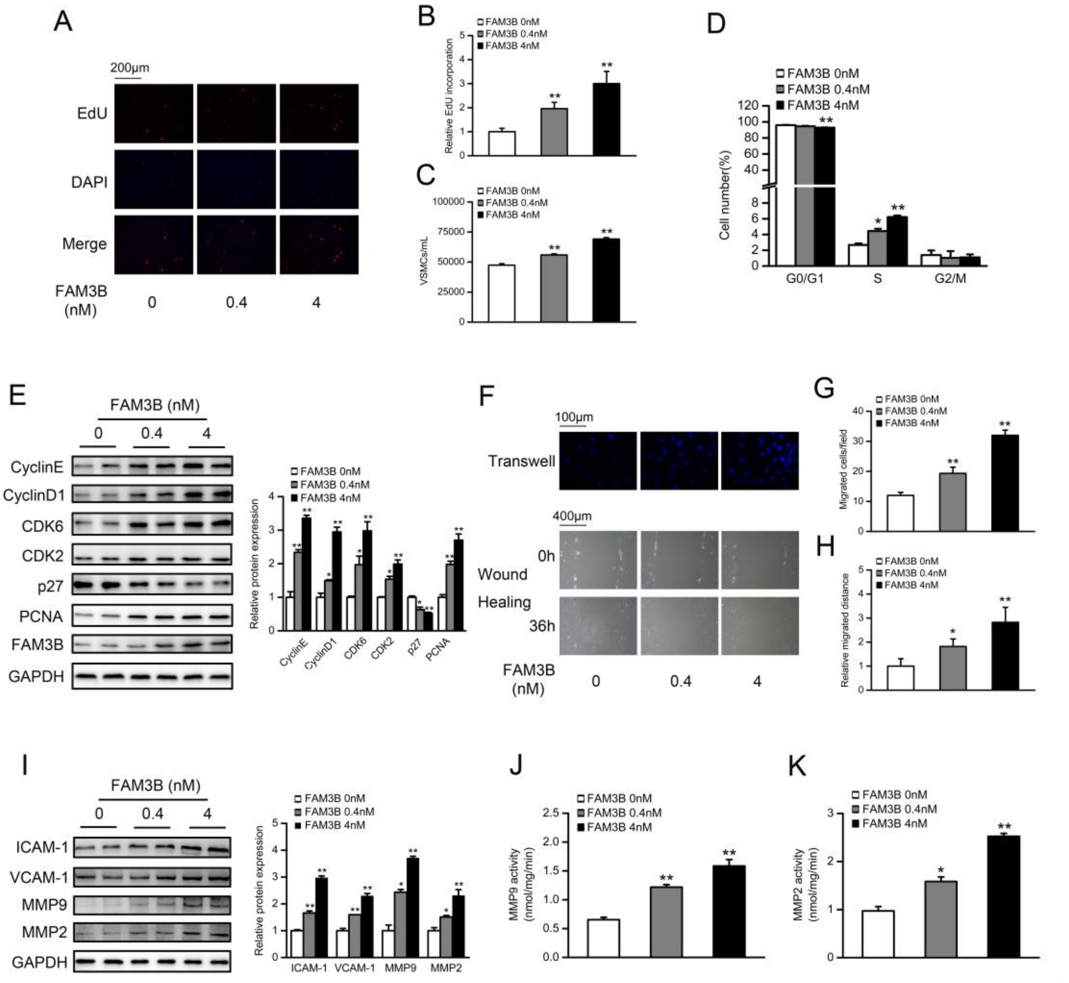


**Figure S7. Stimulation of recombinant FAM3B protein accelerates VSMC proliferation and migration.** VSMCs were treated with recombinant FAM3B protein or equal amount of BSA for 48 h in DMEM medium containing 5.5 mM glucose. Cell proliferation was evaluated using the EdU incorporation (A, B*)* or direct cell counting assay (C). $^{**}P$<0.01 *vs.* FAM3B 0 nM. (D) Assessment of the cell cycle progression by flow cytometry. $^{*}P$<0.05 and $^{**}P$<0.01 *vs.* FAM3B 0 nM. (E) Protein expression levels of key regulators involved in cell cycle progression. $^{*}P$<0.05 and $^{**}P$<0.01 *vs.* FAM3B 0 nM. (F) Determination of VSMC migration by transwell chamber (top) and wound-healing (bottom) assays. (G, H) Quantification of the data from (F). $^{*}P$<0.05 and $^{**}P$<0.01 *vs.* FAM3B 0 nM. (I) Protein expression levels of ICAM-1, VCAM-1, and MMPs. $^{*}P$<0.05 and $^{**}P$<0.01 *vs.* FAM3B 0 nM. (J, K) Total activity of MMP-9 and MMP-2. $^{*}P$<0.05 and $^{**}P$<0.01 *vs.* FAM3B 0 nM.

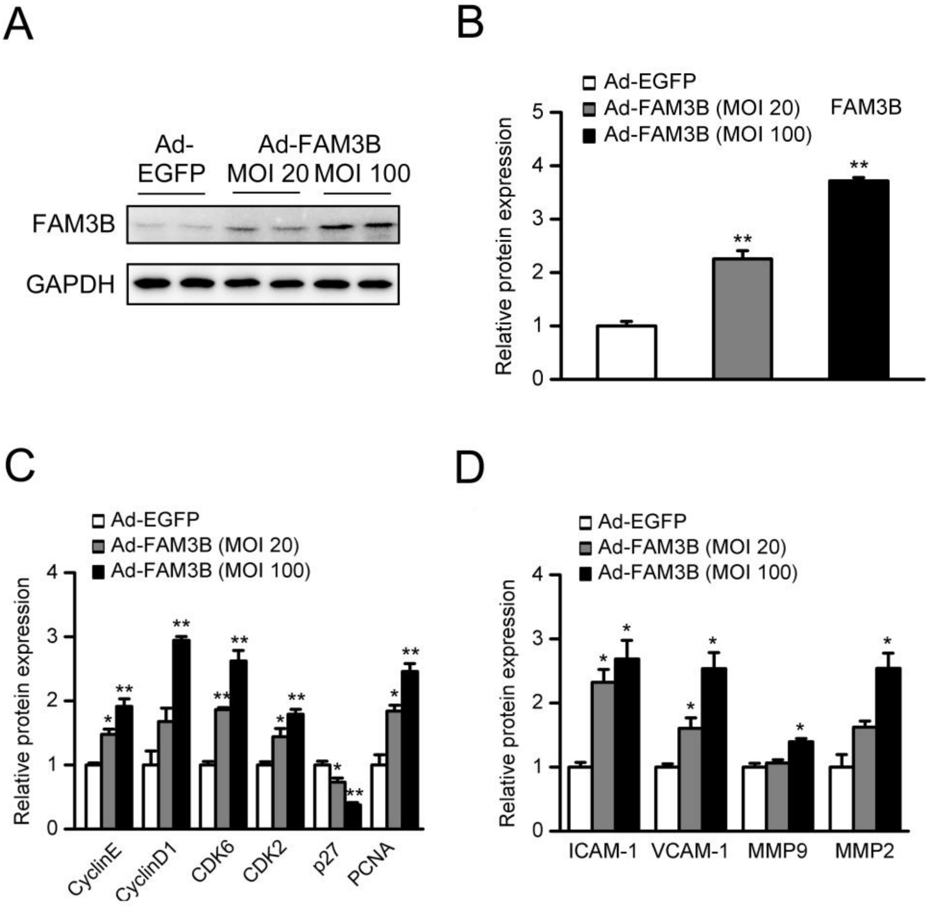


**Figure S8. Overexpression of FAM3B accelerates VSMC proliferation and migration.** (A, B) The efficiency of FAM3B overexpression by adenovirus infection. $^{**}$*P*<0.01 *vs.* Ad-EGFP. (C) Quantification for the immunoblots shown in Fig. 4E. (D) Quantification for the immunoblots shown in Fig. 4I. $^{*}$*P*<0.05 and $^{**}$*P*<0.01 *vs.* Ad-EGFP.

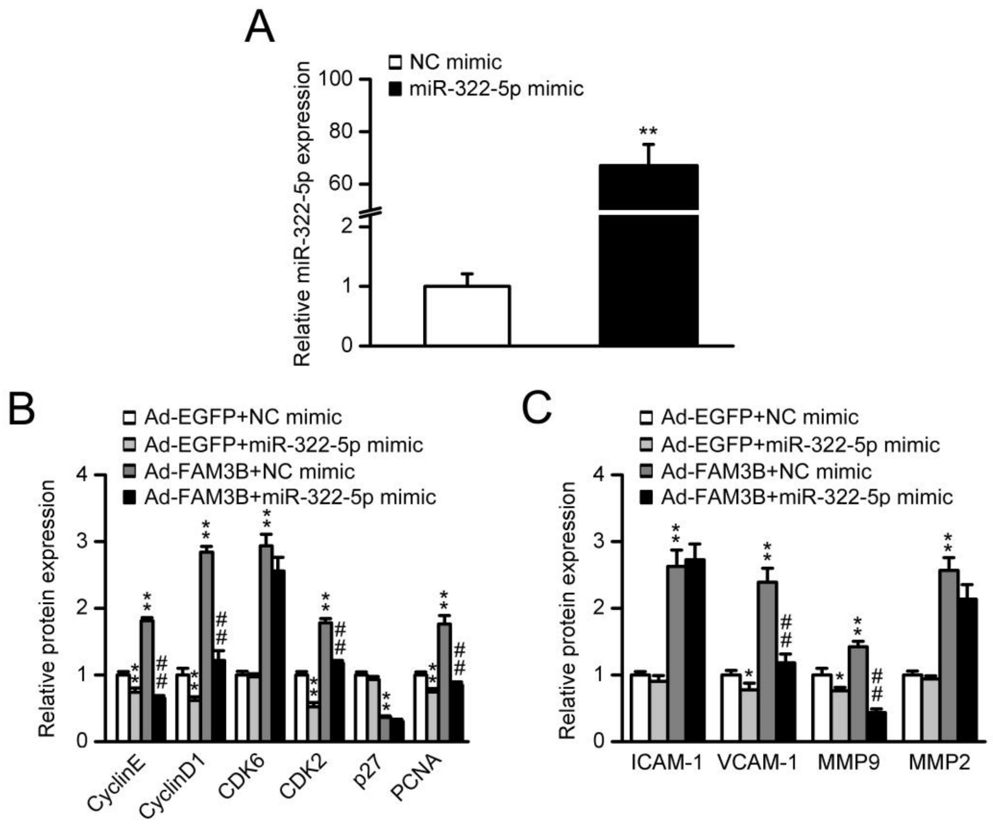


**Figure S9. MiR-322-5p blocks FAM3B-induced expression changes of regulators involved in the cell cycle re-entry and migration.** (A) Validation of the efficiency of miR-322-5p overexpression. $^{**}$*P*<0.01 *vs.* NC mimic. (B) Quantification for the immunoblots shown in Fig. 7C. (C) Quantification for the immunoblots shown in Fig. 7D. $^{*}$*P*<0.05 and $^{**}$*P*<0.01 *vs.* Ad-EGFP/NC mimic, $^{\#\#}$*P*<0.01 *vs.* Ad-FAM3B/NC mimic.
